# Mendelian randomization study reveals the effect of idiopathic pulmonary fibrosis on the risk of erectile dysfunction

**DOI:** 10.3389/fmed.2023.1162153

**Published:** 2023-07-12

**Authors:** Kun Zhang, Jiejun Zhou, Anqi Li, Mingwei Chen

**Affiliations:** Department of Respiratory and Critical Care Medicine, First Affiliated Hospital of Xi'an Jiaotong University, Xi'an, Shaanxi, China

**Keywords:** erectile dysfunction, idiopathic pulmonary fibrosis, Mendelian randomization, GWAS, instrumental variable

## Abstract

**Background:**

Several studies have found that erectile dysfunction (ED) is associated with interstitial lung disease. However, the causal relationship between idiopathic pulmonary fibrosis (IPF) and ED risk remains unclear. The present two-sample Mendelian randomization (MR) study aimed to reveal the causal effect of IPF on ED risk.

**Methods:**

This study included two GWAS summary statistics of IPF (1,028 cases and 196,986 controls) and ED (6,175 cases and 217,630 controls) of European ancestry. The inverse-variance weighted (IVW) was applied as the primary method, and MR-Egger, weighted median, weighted mode, and simple mode were applied as complementary methods to estimate the causal impact of IPF on ED risk. The MR-PRESSO global test and MR-Egger regression were applied to evaluate the pleiotropy. The Cochran’s *Q* test was applied to examine heterogeneity. The leave-one-out analysis ensured the robustness and reliability of the results.

**Results:**

Twenty-one genetic variants were obtained as IPF instrumental variables without pleiotropy and heterogeneity. MR analysis using the IVW showed a potential causal relationship between IPF and increased ED risk (OR_IVW_ = 1.046, 95% CI: 1.020–1.073, *p* = 0.001), and consistent results were obtained with MR-Egger, weighted median, and weighted mode. The leave-one-out analysis showed that no instrumental variables unduly influenced the results.

**Conclusion:**

This study suggested that IPF may increase the ED risk of the European population.

## 1. Introduction

Sexual health is a marker of overall health and good quality of life, of which erectile dysfunction (ED) has gained widespread attention worldwide (1). ED is defined as failing to achieve or sustain a penile erection sufficient for sexual intercourse (2). Current research on the prevalence of ED has found that it is a pervasive problem and increases with age. One survey found that the percentage of men aged under 40 consulting ED increased from 5% in 2010 to more than 15% in 2015 (3). Moreover, 52% of men aged 40–70 had some degree of ED (4). ED imposes a heavy burden on men and has become a health problem that cannot be ignored (5). As a result, it’s essential to discover ED risk factors and evaluate individuals who may need early intervention or prevention.

At the onset of ED, various associated factors have been identified, such as depression, hypertension, diabetes, obesity, and smoking (6–9). Several observational studies have found that ED is a common problem in patients with interstitial lung disease (ILD), especially in idiopathic pulmonary fibrosis (IPF) patients (10, 11). IPF is a chronic fibrotic interstitial pneumonia marked by dyspnea and a gradual decline in lung function (12). Some studies have found that IPF is often combined with chronic hypoxia, which seems to have a shared pathogenic basis with ED (11). Considering that the causal link between IPF and ED risk remains unclear and that observational studies may be influenced by confounding factors or inverse causality to draw unreliable results, further clarification of the causal relationship between IPF and ED risk is necessary.

Two-sample Mendelian randomization (MR) examines the causal link between exposure and outcome by extracting genetic variants as instrumental variables (IVs) (13). Because genetic variants precede disease onset, MR analysis avoids reverse causality and the effects of unmeasured confounders (14), which overcomes the shortcomings present in observational studies. The present two-sample MR study aimed to estimate the causal effect of IPF on ED risk.

## 2. Materials and methods

### 2.1. Ethics statement

Our analyses used summary data from published studies or available genome-wide association studies (GWAS) and did not require ethics committee approval. The institutional ethics review board corresponding to GWAS approved each research, and all subjects signed informed consent.

### 2.2. Study design

**Figure 1:**
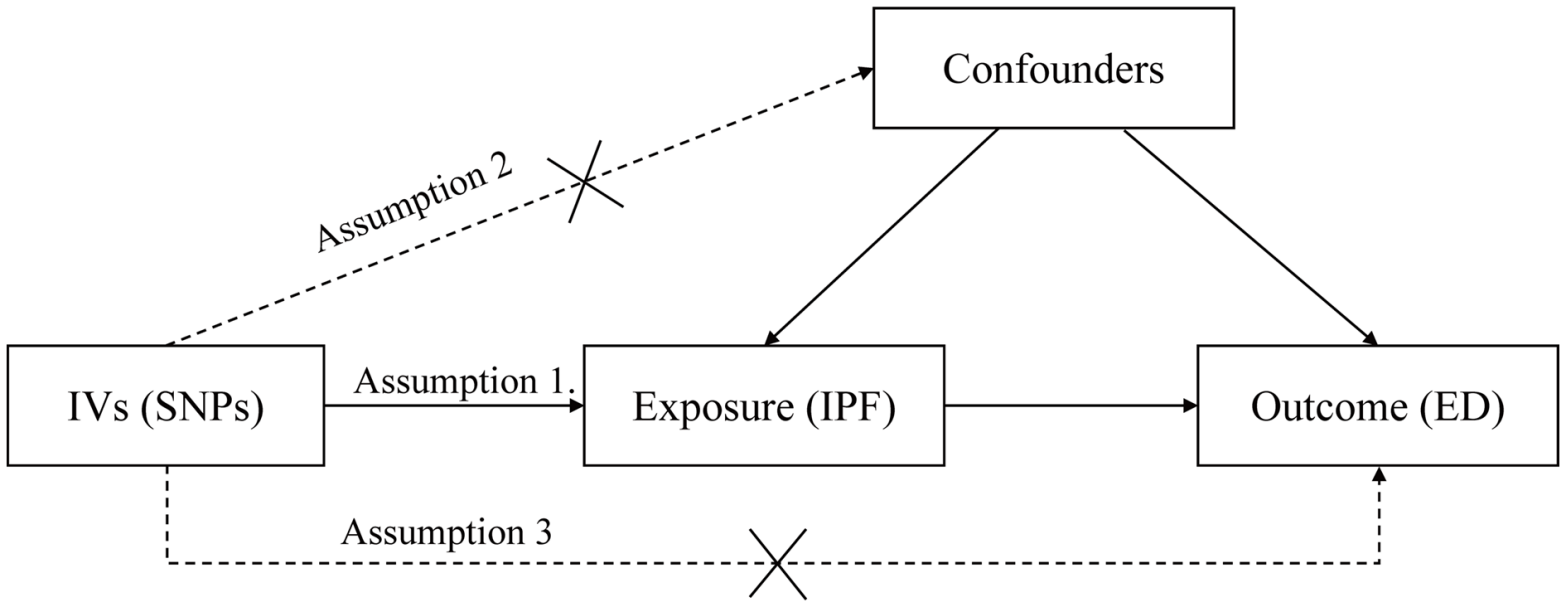
Three assumptions for IVs in MR analysis. ED, erectile dysfunction; IPF, idiopathic pulmonary fibrosis; MR, Mendelian randomization; IV, instrumental variable; SNP, single-nucleotide polymorphism.

Three critical assumptions of the MR study are illustrated in Figure 1. Assumption 1 is that IVs are reliably correlated with IPF. Assumption 2 is that IVs must be independent of any confounders. Assumption 3 is that IVs affect ED risk only through IPF but not through other pathways (14).

### 2.3. Data source

To identify genetic variants associated with IPF, we used the IPF GWAS study in the FinnGen Biobank (1,028 cases and 196,986 controls) (15). To avoid overlap in exposure and outcome populations, we used the summary data from a publicly available GWAS study of ED with populations primarily from UK Biobank, the Partners HealthCare Biobank cohort, and the Estonian Genome Center of the University of Tartu cohorts, including 6,175 ED patients and 217,630 controls (16). Both GWAS subjects are of European ancestry. The IEU open GWAS database (17)^1^ provides the two GWAS summary data (IPF ID: finn-b-IPF; ED ID: ebi-a-GCST006956).

### 2.4. Selection of IVs

We performed strict quality control to obtain qualified instrumental single-nucleotide polymorphisms (SNPs) from the IPF GWAS study. First, based on assumption 1, we extracted SNPs significantly related to IPF (*p* < 5 × 10^−6^) (18). Second, to keep all IVs of the IPF from being in a linkage disequilibrium (LD) state, the clumping parameter is set as *R*^2^ < 0.001 and window size = 10,000 kb (19). Third, we excluded SNPs with minor allele frequencies of less than 0.01. Fourth, we extracted the chosen SNPs from the ED GWAS. If an instrumental SNP does not exist in ED GWAS, we look for a SNP that is in LD status with it instead. Fifth, the harmonization process removed the palindromic SNPs from the IVs. Sixth, to ensure that IVs affect ED risk only through IPF, we examined and removed SNPs in IVs associated with possible confounders such as diabetes, obesity, hypertension, smoking, and metabolic syndrome through the PhenoScanner V2 database (20, 21). In addition, to avoid bias from weak IVs, we calculated the *F*-statistic (*F* = Beta^2^/SE^2^) (22). If the F-statistic of IVs is much larger than 10, the possibility of bias from weak IVs is slight (23).

### 2.5. MR analysis

In the study, we estimated the causal relationship between IPF and ED risk using different methods, including the inverse-variance weighted (IVW) (24), MR-Egger (25), weighted median (26), weighted mode (27), and simple mode (28). These methods have different priorities under different conditions. The IVW method combines SNPs’ Wald estimators to assess the impact of IPF on ED risk. When no pleiotropy exists for IVs or balanced pleiotropy exists, we acquire reliable causal estimates primarily through the IVW approach. If there was significant heterogeneity in the IVs (*p* < 0.05), we utilized a random effect model. Otherwise, a fixed effect model was utilized (19). The MR-Egger regression approach yields reliable estimates when pleiotropy exists for IVs (29). The weighted median approach still yields causal estimates when less than 50% of the IVs contravene the critical MR assumptions (26). The weighted model approach can perform MR causal inference when most IVs are valid (27). The simple mode is less potent than IVW (30). MR analysis was carried out in RStudio 4.2.1 using the R package TwoSampleMR (version 0.5.6) (31). *p* < 0.05 was considered significant.

### 2.6. Pleiotropy, heterogeneity, and sensitivity analysis

The MR-PRESSO global test (32) and MR-Egger regression (25) were utilized to evaluate the pleiotropy of IVs, and IVs were considered to have pleiotropy when *p* < 0.05. Heterogeneity was quantified by the Cochran’s *Q* statistic and deemed significant when *p* < 0.05 (33). Additionally, to determine the presence of SNPs with bias effects, we conducted the leave-one-out analysis (34), which ensured the stability of our results.

## 3. Results

### 3.1. Selection of IVs

According to the screening criteria of the instrumental SNPs, we obtained 21 LD-independent SNPs from the GWAS of IPF (Supplementary Table S1). None of these 21 SNPs were associated with possible confounders of ED, such as diabetes, obesity, hypertension, smoking, and metabolic syndrome. Moreover, all of these 21 SNPs could be extracted in the GWAS of ED (Supplementary Table S2). Ultimately, we obtained these 21 SNPs as IVs of the IPF with *F* > 10 for each instrumental-exposure association, indicating a low likelihood of weak IV bias (Table 1). In addition, the genes corresponding to each instrumental SNP were shown in Supplementary Table S3.

**Table 1:** IVs for IPF.

SNP	Chr	Pos	Beta	SE	Effect_allele	Other_allele	EAF	F	*p*
rs7583252	2	228,838,054	0.216	0.046	A	G	0.423	21.9	2.92E-06
rs12638862	3	169,477,506	0.262	0.051	G	A	0.283	25.94	3.47E-07
rs9848175	3	8,743,076	0.808	0.176	G	A	0.021	21.12	4.35E-06
rs6847916	4	63,661,051	0.766	0.158	G	A	0.023	23.64	1.16E-06
rs558341636	5	1,584,923	2.210	0.365	C	T	0.007	36.71	1.37E-09
rs113548226	5	146,708,779	1.770	0.380	A	T	0.005	21.64	3.29E-06
rs145315307	5	168,904,005	1.019	0.129	G	T	0.037	62.84	2.20E-15
rs80236851	5	52,800,060	0.971	0.187	G	A	0.018	26.85	2.20E-07
rs10069690	5	1,279,790	−0.250	0.050	T	C	0.295	24.76	6.42E-07
rs79479138	5	1,238,988	1.037	0.186	T	C	0.018	30.99	2.59E-08
rs2076295	6	7,563,232	0.215	0.047	G	T	0.383	20.82	4.96E-06
rs116515165	6	2,392,366	0.958	0.208	T	C	0.014	21.23	4.06E-06
rs35000338	11	1,805,022	0.259	0.054	C	G	0.245	23.26	1.39E-06
rs11222805	11	99,979,997	−0.370	0.078	C	A	0.101	22.61	1.97E-06
rs11246335	11	866,133	0.784	0.123	A	G	0.041	40.54	1.91E-10
rs35705950	11	1,241,221	1.605	0.085	T	G	0.103	359.1	3.88E-80
rs72843931	11	1,875,886	0.899	0.132	T	C	0.036	46.47	9.29E-12
rs12605893	18	10,439,563	−0.216	0.046	A	C	0.464	22.32	2.32E-06
rs6423444	20	62,272,411	0.247	0.051	A	G	0.285	23.44	1.27E-06
rs9979837	21	15,359,048	0.227	0.048	T	C	0.363	22.4	2.17E-06
rs192474413	22	44,625,792	0.634	0.136	C	T	0.031	21.69	3.19E-06

IVs, instrumental variables; IPF, idiopathic pulmonary fibrosis; Chr, chromosome; SE, standard errors; EAF, effect allele frequency; Pos, position; SNP, single-nucleotide polymorphism.

### 3.2. Pleiotropy test

We used the MR-Egger regression and MR-PRESSO global test to detect the pleiotropy of IVs. The MR-Egger regression suggested that IVs have no pleiotropy (*p* = 0.064, Table 2), which was confirmed by the MR-PRESSO global test (*p* = 0.600, Table 2). Therefore, our IVs are unlikely to be associated with confounders, and we validated this result in the PhenoScanner V2 database. Therefore, all these 21 IPF genetic variants can be used as valid IVs.

**Table 2:** Pleiotropy test of MR.

Method	Effect size	*p*
MR-Egger regression	−0.020 (egger_intercept)	0.064
MR-PRESSO global test	19.707 (RSSobs)	0.600

MR, Mendelian randomization; RSS, residual sum of squares.

### 3.3. Heterogeneity test

The Cochran’s Q test was used to detect heterogeneity in IVs. The Cochran’s *Q* statistic suggested that IVs have no heterogeneity (*P*_IVW_ = 0.549, *P*_MR-Egger_ = 0.741, Table 3). Therefore, we primarily use the fixed effect model to perform MR causal estimates.

**Table 3:** Heterogeneity test of MR.

Method	Q	Q_df	*p*
MR-Egger	14.713	19	0.741
Inverse-variance weighted	18.588	20	0.549

MR, Mendelian randomization.

### 3.4. MR estimates

**Figure 2:**
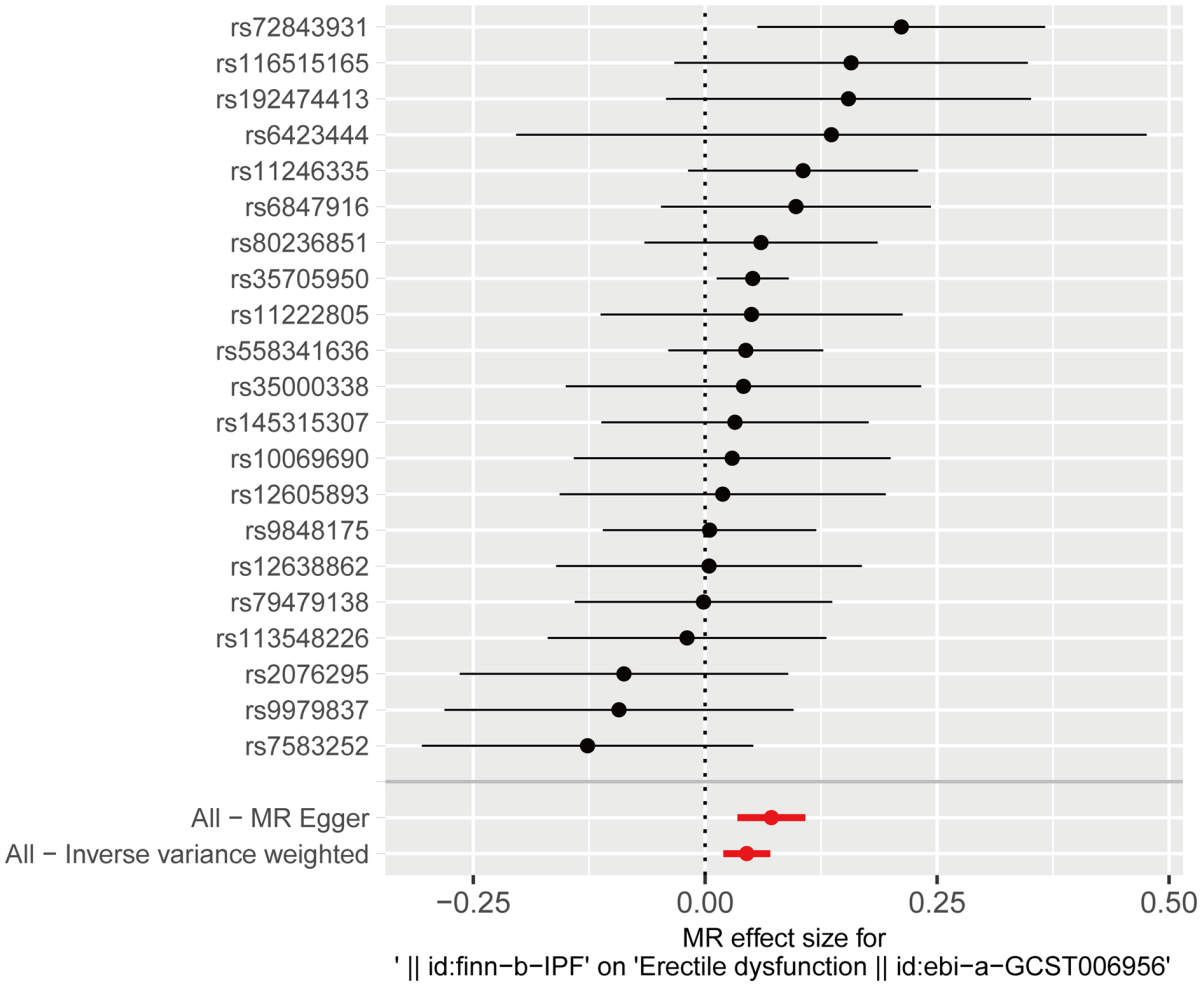
Forest plot for the causal effect of each SNP on ED risk. ED, erectile dysfunction; SNP, single-nucleotide polymorphism.

**Figure 3:**
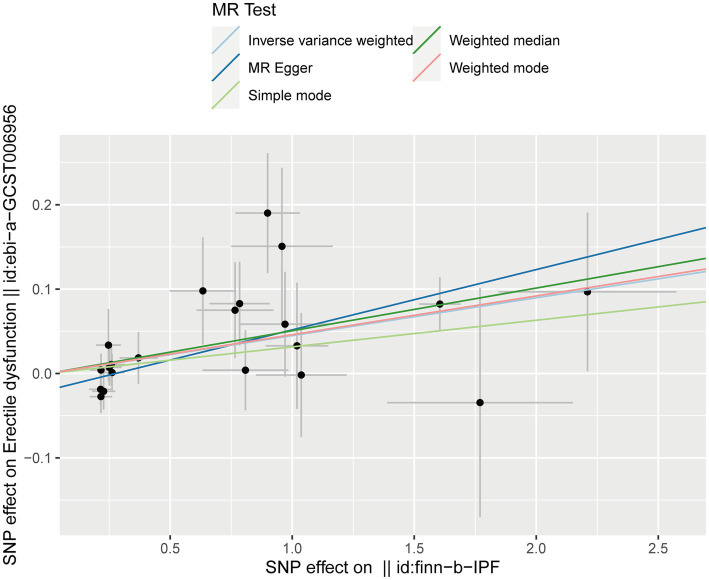
Scatter plot for the causal effect of IPF on ED risk. ED, erectile dysfunction; IPF, idiopathic pulmonary fibrosis.

After testing for pleiotropy and heterogeneity, we obtained 21 SNPs as IVs to assess the genetic association of IPF and ED risk, and the forest plot displays each SNP’s causal impact on ED (Figure 2). Table 4 displayed the MR effect sizes for various approaches to evaluating the causal impact of IPF on ED risk. The IVW result revealed a causality of IPF on ED risk (OR = 1.046, 95% CI: 1.020–1.073, *p* = 0.001). Also, MR-Egger (OR = 1.074, 95% CI: 1.035–1.114, *p* = 0.001), weighted median (OR = 1.052, 95% CI: 1.017–1.088, *p* = 0.003) and weighted mode (OR = 1.047, 95% CI: 1.010–1.086, *p* = 0.022) approaches also obtained consistent results. In addition, the MR estimates of SNPs on both IPF and ED are shown in the scatter plot (Figure 3).

**Table 4:** Causal effect of IPF on ED risk.

Method	*N* (SNP)	Beta	SE	*p*	OR	OR_lci95	OR_uci95
MR-Egger	21	0.071	0.019	0.001	1.074	1.035	1.114
Weighted median	21	0.051	0.017	0.003	1.052	1.017	1.088
Inverse-variance weighted	21	0.045	0.013	0.001	1.046	1.020	1.073
Simple mode	21	0.032	0.031	0.324	1.032	0.971	1.097
Weighted mode	21	0.046	0.019	0.022	1.047	1.010	1.086

ED, erectile dysfunction; IPF, idiopathic pulmonary fibrosis; SNP, single-nucleotide polymorphism; SE, standard error; OR, odds ratio; OR_lci95, lower limit of 95% confidence interval for OR. OR_uci95, upper limit of 95% confidence interval for OR.

### 3.5. Leave-one-out analysis

**Figure 4:**
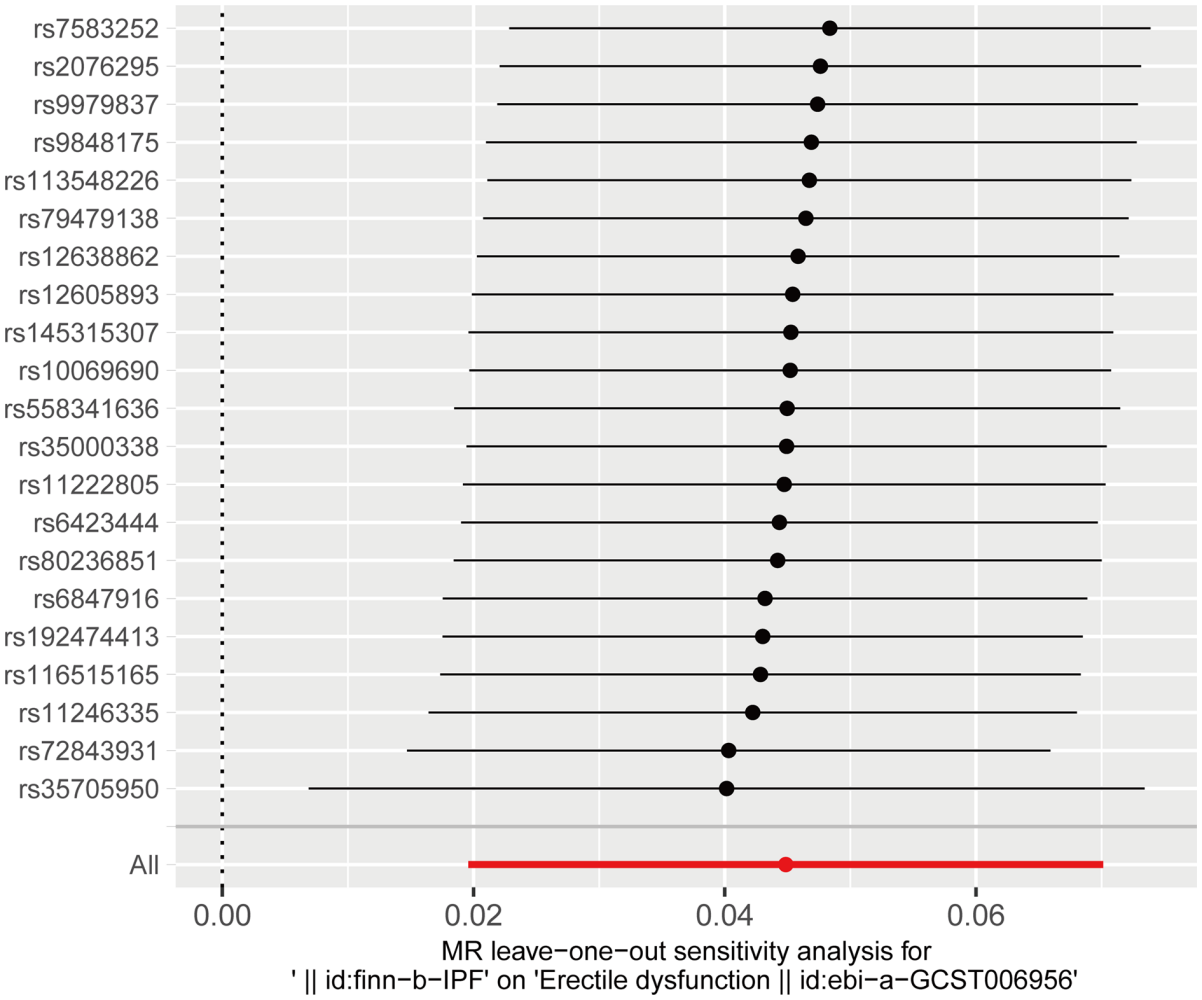
Leave-one-out analysis of the effect of IPF on ED risk. ED, erectile dysfunction; IPF, idiopathic pulmonary fibrosis.

The leave-one-out analysis was performed by excluding each SNP one by one and then observing whether the results changed. The leave-one-out analysis ensures that a particular SNP does not unduly influence the results. The result indicated no SNPs that significantly influenced ED risk and thus biased the causal estimates of MR. Therefore, our results were robust and not significantly biased (Figure 4).

## 4. Discussion

Epidemiological studies have found that ED has a high prevalence in men and increases with age. ED has become an important health issue due to its severe impact on men’s quality of life and psychosocial health (6). Therefore, the study of risk factors for ED is of great value for prevention and early intervention in ED.

Our study used the two-sample MR analysis to determine the causal link between IPF and ED risk. The results suggested that IPF causally increases the risk of ED, which is robust and reliable. A study investigating the prevalence of ED in ILD patients found that ED prevalence was much higher in ILD patients than in the background population and that ED prevalence increased with reduced pulmonary diffusion capacity (11). In another study of eight men with IPF-related hypoxia, nearly all of these men suffered from sex hormone suppression and ED (10). Although all of these studies suggest an association between IPF patients and increased ED risk, none can clarify the causal effects of IPF on ED risk. Moreover, observational studies are vulnerable to confounders or inverse causality. In contrast, our study provides causal evidence of IPF increasing ED risk within the MR design framework.

Although this study established a causal relationship between IPF and ED risk, the mechanisms by which IPF increases ED risk are unclear and may include common disease mechanisms of IPF and ED. Some instrumental SNPs have been shown to be closely associated with IPF, for example, the specific mutation rs35705950 (MUC5B) associated with fibrosis. However, the effect of these IVs on ED risk is via IPF rather than directly associated with ED, and IVs cannot be associated with ED susceptibility factors (confounders), including diabetes, obesity, hypertension, smoking, and metabolic syndrome. So, through what pathways does IPF increase ED risk? A recent study of COPD patients suffering from ED found that hypoxemia may be the mechanism by which COPD patients develop ED (35). In contrast, long-term oxygen therapy reversed ED in patients with hypoxemia (36). And patients with IPF-related hypoxia also developed sex hormone suppression and ED (10). In addition, the progression of IPF was associated with vascular endothelial dysfunction (37), which is characteristic of ED (38). Therefore, vascular endothelial dysfunction and hypoxemia may mediate IPF to increase ED risk.

Notably, our analysis is the first MR study of IPF on ED risk. We selected strongly correlated and independently inherited LD-independent SNPs as IVs to estimate the causal effect of IPF on ED risk. The strength of the link between IVs and IPF was assessed using an F-statistic that was much larger than 10, suggesting a low likelihood of weak IV bias (23). Several robust analysis methods provided robust inferences for our MR analysis. In addition, the current MR analysis used GWAS data from two large samples of European populations, providing sufficient power to estimate causality while avoiding demographic bias. The populations of these two GWAS studies were from two databases, minimizing the possibility of subject overlap, and we minimized the bias of sample overlap by using strong instruments (*F* > 10) (39). Finally, MR analysis revealed the effect of genetically predicted IPF on ED risk, overcoming the shortcomings of observational studies that are susceptible to confounders.

The study also has some limitations. First, the summary data of the GWAS involved only European ancestry, and our conclusions may only apply to European populations. Therefore, we ought to use our findings with caution in racially and ethnically diverse populations. Second, since the exact functions of some SNPs in IVs are not known, there may be residual bias. Furthermore, given the binary assessment of IPF and the lack of individual statistics, it was impossible to explore nonlinear associations between IPF and ED risk, perform stratified analyses, and adjust for other covariates.

## 5. Conclusion

In this study, we showed an apparent causal effect of genetically predicted IPF on ED risk in the European population using a two-sample MR analysis. However, further mechanistic studies are necessary to explain the deeper link between IPF and increased ED risk. Most importantly, our study reminds clinicians that measures to prevent and intervene early in ED should be considered when diagnosing male patients with IPF.

## Data availability statement

The original contributions presented in the study are included in the article/Supplementary material, further inquiries can be directed to the corresponding author.

## Author contributions

KZ and MC conceived the study and wrote the manuscript. KZ, JZ, and AL collected the data and conducted the analysis. All authors contributed to the article and approved the submitted version.

## Conflict of interest

The authors declare that the research was conducted in the absence of any commercial or financial relationships that could be construed as a potential conflict of interest.

## Publisher’s note

All claims expressed in this article are solely those of the authors and do not necessarily represent those of their affiliated organizations, or those of the publisher, the editors and the reviewers. Any product that may be evaluated in this article, or claim that may be made by its manufacturer, is not guaranteed or endorsed by the publisher.

## Footnotes

^1^https://gwas.mrcieu.ac.uk/datasets/
